# Hybridization Design and High-Throughput Screening of Peptides with Immunomodulatory and Antioxidant Activities

**DOI:** 10.3390/ijms26020505

**Published:** 2025-01-09

**Authors:** Junyong Wang, Rijun Zhang, Xuelian Zhao, Jing Zhang, Yucui Tong, Zaheer Abbas, Zhenzhen Li, Haosen Zhang, Dayong Si, Xubiao Wei

**Affiliations:** Laboratory of Feed Biotechnology, State Key Laboratory of Animal Nutrition and Feeding, College of Animal Science and Technology, China Agricultural University, Beijing 100193, China; wangjy9722@163.com (J.W.);

**Keywords:** hybrid peptide, immunomodulatory, antioxidant, molecular docking

## Abstract

With the increasing recognition of the role of immunomodulation and oxidative stress in various diseases, designing peptides with both immunomodulatory and antioxidant activities has emerged as a promising therapeutic strategy. In this study, a hybridization design was applied as a powerful method to obtain multifunctional peptides. A total of 40 peptides with potential immunomodulatory and antioxidant activities were designed and screened. First, molecular docking was employed to screen peptides with a high binding affinity to MD2, a key receptor protein in the NFκB immune pathway. For the in vitro high-throughput screening, we constructed a reporter gene-based stable cell line, IPEC-J2-Lucia ARE cells, which was subsequently used to screen peptides with antioxidant activity. Furthermore, the biocompatibility, immunomodulatory, and antioxidant activities of these peptides were assessed. Among the candidates, the hybrid peptide VA exhibited the strongest immune-enhancing activity through the activation of the NF-κB pathway and significant antioxidant activity via the Nrf2-ARE pathway. Additionally, VA demonstrated protective effects against H_2_O_2_-induced oxidative damage in HepG2 cells. This study not only demonstrates the potential of peptide hybridization, but also develops a screening platform for multifunctional peptides. It provides a new tool for the treatment of autoimmune diseases and oxidative stress-related diseases.

## 1. Introduction

The immune system plays a crucial role in defense against foreign pathogens [1,2,3]. Immunodeficiency or dysregulation of the immune response can lead to a variety of diseases and, in severe cases, to cancer [4,5,6]. Toll-like receptors (TLRs), particularly TLR4/MD2, are key components of the innate immune system and are responsible for pathogen recognition [7]. TLR4/MD2 recognizes agonist molecules (e.g., lipopolysaccharide (LPS)) to initiate immune responses [7,8,9]. Therefore, targeting TLR4/MD2 for immune enhancements is an effective strategy to improve immune defense capacity [10,11,12].

Oxidative stress occurs when the body’s redox homeostasis is disrupted, which is usually due to an excess of reactive oxygen species (ROS) [13]. ROS can damage cellular DNA, proteins, and lipids, leading to cellular dysfunction and diseases such as cancer, atherosclerosis, and rheumatoid arthritis [13,14]. The Keap1-Nrf2-ARE pathway is a major antioxidant defense mechanism [14,15,16]. In response to ROS signaling, Nrf2 dissociates from Keap1 and translocates to the nucleus, where it binds to antioxidant response elements (ARE) and activates the expression of downstream antioxidant genes, thereby restoring redox homeostasis and protecting the cell from damage [16,17]. In recent years, considerable attention has been paid to the development of peptides that can modulate both immune regulation and oxidative stress [10,17,18,19,20]. However, few studies have focused on peptides that target both immune enhancement and antioxidant stress, which could offer more efficient and cost-effective solutions to issues in fields such as medicine, food, and agriculture.

Hybridization design strategies can combine functional fragments of different peptides to create hybrid peptides with multiple functions [21,22]. This approach has been widely applied in various research studies due to its simplicity and efficiency [23,24]. In addition to peptide design, another major challenge is identifying functional peptide fragments from large peptide sequence libraries. With the rapid development of computer technology, computational-based virtual screening has made the high-throughput screening of functional peptides possible [25,26]. Molecular docking, a powerful screening tool, allows for the efficient identification of peptide sequences with high binding affinity to target proteins, thus greatly reducing the workload of screening potential candidates [11,27,28]. There are several protein–peptide docking software tools available, such as ROSETTA FlexPepDock [29], ZDOCK [30], AUTO DOCK [31], HDOCK [32], and HADDOCK peptide [33]. Each docking software has its own algorithmic preferences that may yield a different performance depending on the specific task. HDOCK outperforms most docking software in global rigid-body blind docking, particularly in handling large-scale docking tasks, where it provides higher accuracy and efficiency. More importantly, HDOCK’s docking speed is so fast that the average processing time per project is less than 30 min [32]. This is especially important for rapid screening in drug development and molecular design. On the other hand, cell-based screening models utilizing luciferase reporter genes have greatly improved the efficiency of high-throughput drug screening [34,35,36]. Unlike virtual screening, cell-based assays ensure that potential bioactive targets are not overlooked. Additionally, the results obtained from cell-based assays are more convincing and reliable.

**Figure 1:**
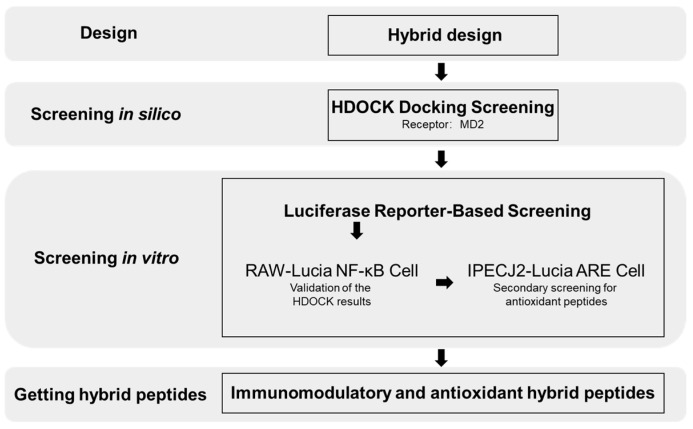
Workflow for the design and screening of immunomodulatory and antioxidant hybrid peptides.

This study aims to design hybrid peptides that simultaneously target MD2 for immune regulation and the Nrf2-ARE pathway for antioxidant stress (Figure 1). Based on our previous research and the literature, we selected nine peptides as parent sequences for hybrid peptide design [22,37,38,39,40,41,42]. According to the results of our preliminary experiments, these peptides were divided into two groups: immune-modulatory peptides (P, Ta, Tb, A) and antioxidant peptides (R, H, Y, M, V) (Figure S1). Subsequently, 40 hybrid peptides were obtained through hybrid design. Through molecular docking, we identified potential immune-enhancing peptides that interact with MD2. Additionally, we used an ARE-based reporter gene assay to screen for peptides with antioxidant activity. By combining these two functions, we aim to discover hybrid peptides that both enhance immune responses and protect against oxidative stress, offering new therapeutic strategies for diseases associated with immunodeficiency and redox imbalance.

## 2. Results

### 2.1. Hybrid Peptide Design

Nine peptides with potential immunomodulatory or antioxidant activities were selected as parent peptides. The sequences of these parent peptides are listed in Table 1: TP5 (P), Tα1-a (Ta), Tα-b (Tb), Aβ (A), RP (R), PH (H), YG9 (Y), MMO (M), and VLP (V). Based on our preliminary experiments, the parent peptides were categorized into two groups: those with potential immunomodulatory activity (P, Ta, Tb, A) and those with antioxidant activity (R, H, Y, M, V). By hybridizing peptides from the two groups, a total of 40 designed hybrid peptides were obtained (Table 2).

### 2.2. Hybrid Peptide Sequence Information and Toxicity Prediction

Forty hybrid peptides were designed, and their sequence information, molecular weights, solubility, and toxicity profiles were summarized (Table 2). The length of the peptides ranged from 10 residues (PR) to 52 residues (AH) and the predicted molecular weights ranged from 1184.35 Da to 5800.44 Da. The solubility of all designed hybrid peptides was predicted to be “Good”, and toxicity predictions indicated that none of the peptides were toxic. These results suggest that the hybrid peptides do not pose significant biosafety concerns and are suitable for subsequent molecular docking screenings.

### 2.3. Three-Dimensional Modeling and Molecular Docking with HDOCK

**Figure 2:**
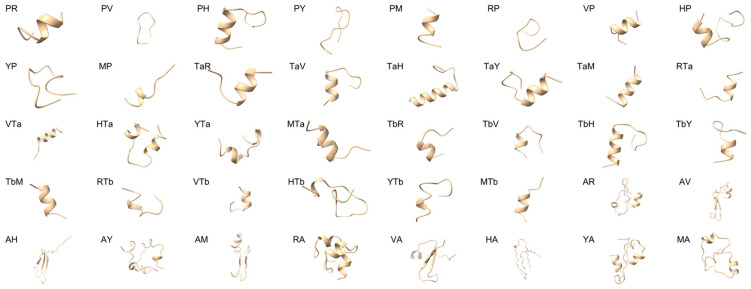
Three-dimensional structural modeling of 40 hybrid peptides using PEPFOLD 3.5.

The three-dimensional structures of all 40 hybrid peptides were predicted using PEPFOLD 3.5, generating 200 different models for each peptide. The top-ranked model for each peptide was selected for further docking studies and the predicted structures are shown in Figure 2. After obtaining the 3D structures of the hybrid peptides, global rigid docking simulations of the hybrid peptide were performed using HDOCK with the MD2 crystal structure (PDB ID: 2Z64) as the receptor. Each peptide was docked with MD2 for 4000 global random docking simulations, and the top-scoring docking pose for each peptide was selected as its optimal docking conformation with MD2. The 40 peptides were ranked according to their docking scores (Table 3). The hybrid peptide YP exhibited the highest HDOCK score of −309.2, while the lowest score was observed for RA (−131.3). Further structural analysis using Ramachandran plots confirmed that the docking complexes of all 40 peptides had high structural validity, with more than 90% of amino acid residues being located in the core+allowed region, indicating that the docking complex adheres to stereochemical conformational rules and is reasonable (Table 4). These results suggest that the docking complexes are valid and can be used for subsequent analysis. The top ten peptides based on docking scores were selected as candidates for further in vitro functional validation.

### 2.4. Biocompatibility of Hybrid Peptides

**Figure 3:**
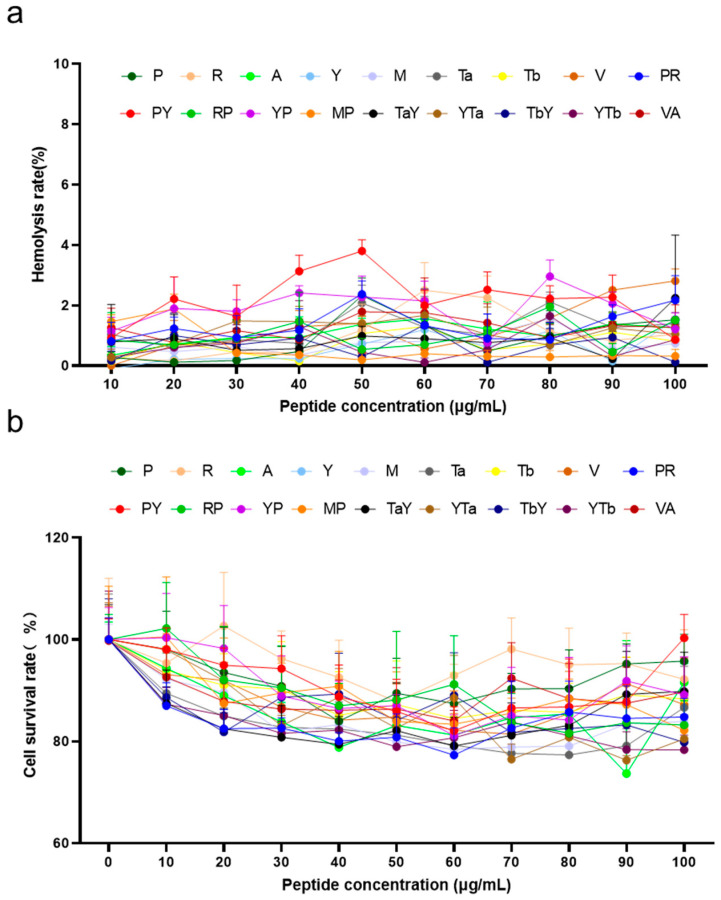
Biocompatibility evaluation of candidate hybrid peptides and their parent peptides. (**a**) Hemolytic activity of hybrid peptides and their parent peptides on sheep red blood cells. (**b**) Cytotoxicity of hybrid peptides and their parent peptides on RAW 264.7 cells. The data are presented as the mean ± SD (n = 3).

The biocompatibility of the chemically synthesized hybrid peptides was evaluated through hemolysis and cytotoxicity assays. The hemolysis assay results showed that the hemolysis rate of the ten candidate hybrid peptides and their corresponding parent peptides was less than 5% within the concentration range of 0–100 μg/mL (Figure 3a), indicating no significant hemolytic activity. The cytotoxicity of all candidate hybrid peptides in the range of 0–100 μg/mL was less than 20% (Figure 3b). These findings are consistent with the predicted biosafety results, further confirming the reliability of the computational predictions.

### 2.5. Screening Hybrid Peptides for Immune Modulatory and Antioxidant Activities Using Luciferase Reporter Assays

**Figure 4:**
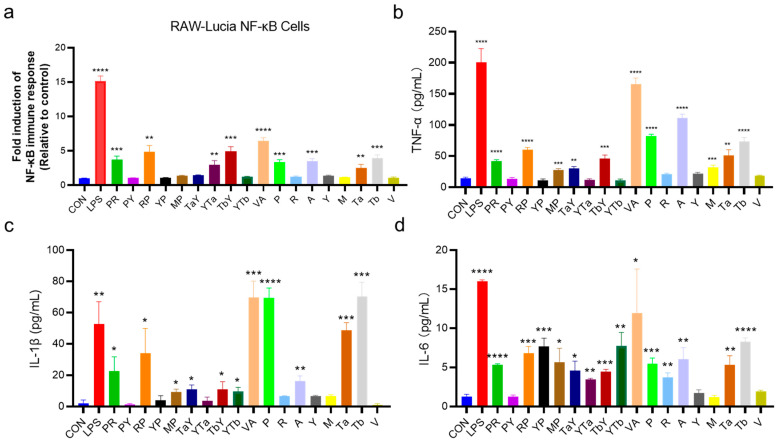
Immunomodulatory activity of candidate hybrid peptides. (**a**) Level of activation of RAW-Lucia NF-κB cells by hybrid peptides and their parent peptides. (**b**–**d**) Effect of hybrid peptides and their parent peptides on the secretion levels of immune-modulatory factors TNF-α (**b**), IL-1β (**c**), and IL-6 (**d**) in RAW 264.7 cells. The data are presented as the mean ± SD (n = 3). * *p* ≤ 0.05, ** *p* ≤ 0.01, *** *p* ≤ 0.001, and **** *p* ≤ 0.0001 compared to the CON group.

**Figure 5:**
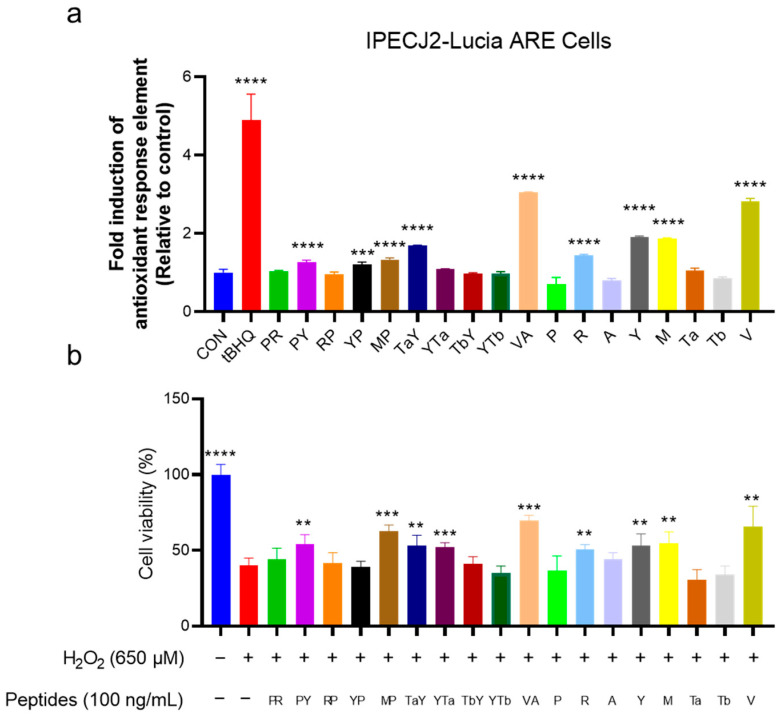
Screening and verification of the antioxidant activity of candidate hybrid peptides. (**a**) Stimulation activity of candidate hybrid peptides and their parent peptides on IPEC-J2-Lucia ARE cells. *** *p* ≤ 0.001 and **** *p* ≤ 0.0001 compared to the CON group. (**b**) Protective effect of candidate hybrid peptides and their parent peptides on H_2_O_2_-induced oxidative damage in the HepG2 model. ** *p* ≤ 0.01, *** *p* ≤ 0.001, and **** *p* ≤ 0.0001 compared to the H_2_O_2_-treated group.

The immunomodulatory and antioxidant activities of the candidate hybrid peptides were screened using RAW-Lucia NF-κB and IPEC-J2-Lucia ARE cell lines, which were constructed in our laboratory. Figure 4a shows the results of the peptide activation of the NF-κB signaling pathway. The results indicated that hybrid peptides PR, RP, YTa, TbY, and VA significantly activated the NF-κB signaling pathway (*p* < 0.05), with VA showing the strongest activation; the activation level was increased 6.5-fold compared to the control group (Figure 4a). In terms of antioxidant activity, the hybrid peptides PY, YP, MP, TaY, and VA were found to stimulate the ARE pathway (Figure 5a). VA exhibited the highest ARE activation, with a three-fold increase compared to the control group. Taken together, these results demonstrate that the hybrid peptide VA possesses both immune-modulatory and antioxidant activities.

### 2.6. Immune-Enhancing Activity of Hybrid Peptides

The immune-modulatory effects of the hybrid peptides were evaluated by incubating them with RAW 264.7 mouse macrophage cells for 24 h, followed by measurement of the expression levels of immune-regulatory factors TNF-α, IL-6, and IL-1β (Figure 4b–d). The results showed that hybrid peptides PR, RP, MP, TaY, TbY, and VA significantly upregulated the expression of TNF-α, IL-6, and IL-1β. YTb particularly upregulated IL-6 and IL-1β, while YTa only significantly increased IL-6 expression. These results are consistent with those obtained from the RAW-Lucia NF-κB assay (Figure 4a), indicating that the in vitro screening and validation of immunomodulatory peptides using RAW-Lucia NF-κB is feasible.

### 2.7. Protective Effect of Hybrid Peptides Against H_2_O_2_-Induced Oxidative Damage in HepG2 Cells

To further assess the antioxidant activity of the hybrid peptides, we employed a hydrogen peroxide (H_2_O_2_)-induced oxidative damage model in HepG2 cells. The results are shown in Figure 5b. After 6 h of H_2_O_2_ treatment, cell viability dropped below 50%. However, pre-incubation with certain hybrid peptides significantly enhanced cell survival and alleviated H_2_O_2_-induced cell damage. Hybrid peptide treatment groups that showed significant differences compared to the model group included PY, MP, TaY, YTa, and VA. Among them, VA exhibited the strongest protective effect, increasing HepG2 cell viability by 30% compared to the H_2_O_2_-treated group (Figure 5b). Moreover, the weakest peptide (YTa) still resulted in a 12% increase in cell survival compared to the model group.

## 3. Discussion

Both immunomodulatory and antioxidant stress agents play vital roles in the prevention and treatment of various diseases [6,15,43,44]. In recent years, significant research has been dedicated to these two areas, with peptide-based drugs gaining considerable attention due to their high efficacy and low toxicity [45,46]. However, most peptide drug development has, thus far, targeted only one of these functions—either immunomodulatory or antioxidant stress—while peptides that combine both functions remain rare [18,20,44,47]. The aim of this study was to develop a peptide drug with both immunomodulatory and antioxidant properties.

Peptide hybridization represents an efficient and rapid approach for designing bifunctional or multifunctional peptides [21,23,24]. This method has been successfully used to generate peptides with dual or even multiple functions, providing promising therapeutic candidates and making the hypothesis of this study feasible [22]. In this study, based on a literature review and prior work in our laboratory, we selected nine peptides with immunomodulatory or antioxidant properties. These peptides were divided into two groups: the immunomodulatory peptide group (P, A, Ta, Tb) and the antioxidant stress peptide group (R, Y, M, V, H) (Table 1). Through peptide hybridization, we synthesized 40 hybrid peptides to screen for bifunctional hybrid peptides in this study (Table 2).

Traditionally, peptides are chemically synthesized and then tested for their functionality in vitro [47,48]. However, this method is not optimal for large-scale peptide screening due to the high costs associated with peptide synthesis. Recent advances in computational methods, such as virtual screening and molecular docking, have greatly enhanced the efficiency of drug discovery by enabling in silico screening [25,29,32]. These methods provide a cost-effective alternative.

The TLR4/MD2 complex is an important component of the pattern recognition receptor family, playing a critical role in the recognition of lipopolysaccharides (LPS) from Gram-negative bacteria [7,49]. Additionally, TLR4/MD2 is a valuable target for immunomodulatory agents, as it can activate the immune system and enhance cellular immune responses [9]. In the process of LPS recognition, the activation of TLR4 is dependent on the binding of LPS to MD2 [7,50]. This binding leads to a conformational change in TLR4, transducing extracellular signals into the cell and triggering a cascade of intracellular events [7,50]. Many immune-boosting agents targeting the TLR4-NF-κB pathway work through this mechanism, with drugs binding to MD2 to activate TLR4 [11,34,51]. Based on this principle, we used the crystal structure of MD2 as the receptor in molecular docking simulations, with the designed hybrid peptides being used as ligands. Using the HDOCK platform, we performed a global blind docking to analyze the potential binding modes of the hybrid peptides to MD2.

From the docking results, we selected the top 10 hybrid peptides, which were chemically synthesized for functional validation using the RAW-Lucia NF-κB reporter cell model. The results showed that five of these hybrid peptides (PR, RP, YTa, TbY, and VA) successfully enhanced the NF-κB pathway (Figure 4a). Furthermore, by measuring the expression of immune-regulatory cytokines (TNF-α, IL-6, and IL-1β) in RAW 264.7 cells treated with the hybrid peptides, we confirmed that these peptides promoted the expression of immune-regulatory factors (Figure 4b–d). These findings highlight the effectiveness of computational screening in identifying potential immunomodulatory peptides, while emphasizing the importance of experimental validation.

The Nrf2-ARE pathway is a key signaling pathway in the cellular response to oxidative stress, playing a crucial role in protecting cells against oxidative damage [13,14]. The Antioxidant Response Element (ARE, 5′-(A/G)TGACNNNGC(A/G)-3′) is a conserved sequence found in the promoter regions of detoxification and antioxidant genes; Nrf2 regulates the expression of these genes by binding to the ARE [14,35]. This activation leads to the transcription of genes such as NQO1, GST, GSH, and HO1, which are involved in cellular protection against harmful environments [16]. Therefore, by measuring the activation intensity of the ARE reporter gene, the activation level of the Nrf2-ARE signaling pathway by the test drugs can be indirectly assessed, providing further insights for research [35,52]. In this study, we constructed the IPEC-J2-Lucia ARE cell model to screen the candidate hybrid peptides’ ability to activate the ARE pathway. The results revealed that five of the hybrid peptides (PY, YP, MP, TaY, and VA) significantly activated ARE, with VA demonstrating the most potent antioxidant activity, increasing ARE activation threefold compared to the control group (Figure 5a). Meanwhile, we performed a secondary validation of the screening results using the H_2_O_2_-induced HepG2 oxidative damage model (Figure 5b). The results showed that PY, MP, TaY, and VA were consistent with the previous findings, further confirming the stability and reliability of the screening cell line we established.

Based on our designed screening process for immunomodulatory and antioxidative hybrid peptides, we ultimately identified a peptide, VA, that possesses both functions. Cell-based assays were conducted to validate the activity of VA, further confirming the feasibility of our hybrid peptide screening strategy. Additionally, many studies have been conducted using virtual screening based on molecular docking. However, most studies focus on screening from peptide libraries, with no specific functional targets [10,53]. For example, 519 peptides were derived from gelatin hydrolysates, and through molecular docking, only two peptides capable of binding to TLR4/MD2 were identified [10]. The efficiency of obtaining useful peptide sequences using this screening model is relatively low.

In contrast, using the approach designed in this study, we successfully obtained a hybrid peptide, VA, with both immune-modulatory and antioxidant activities, suggesting that our design and screening method can enhance the efficiency of obtaining functional peptides. However, as the current number of tests is relatively small, further large-scale experiments are necessary to fully validate the reliability and efficiency of our approach.

Moreover, our approach has certain limitations. It relies on accumulated experience and validation to select parent peptide sequences. Additionally, the activity of parent peptides from different sources can vary, making it challenging to achieve the desired outcomes in subsequent design and screening processes, especially when the initial activity of the parent peptide is weak. An example of this is the antioxidant parent peptide R (Figure 5). Therefore, an important focus for optimizing our approach in future work will be determining how to efficiently identify source peptides with strong biological activity.

## 4. Materials and Methods

### 4.1. Peptide Design and Synthesis

Parent peptides with potential immunomodulatory and antioxidant activities were identified, and hybrid peptides were constructed by combining the parent peptides. Details of the hybrid and parent peptides are provided in Table 1 and Table 2. The molecular weight and solubility of the peptides were predicted using the online tool provided by NovoPro (https://www.novopro.cn/tools/calc_peptide_property.html, (accessed on 7 January 2025)) (Shanghai, China). Toxicity predictions were made using the ToxinPred online tool (https://webs.iiitd.edu.in/raghava/toxinpred/, (accessed on 7 January 2025)). The hybrid peptides and corresponding parent peptides were synthesized by GL Biochem Ltd. with a purity of 95% (Shanghai, China). The molecular masses of the peptides were confirmed using MALDI-TOF-MS, and the data are provided in the Supplementary Materials.

### 4.2. Peptide Modeling and Molecular Docking

The 3D structures of all designed peptides were modeled using the PEP-FOLD 3.5 web server (https://mobyle.rpbs.univ-paris-diderot.fr/cgi-bin/portal.py#forms::PEP-FOLD3, (accessed on 7 January 2025)). The crystal structure of MD2 was retrieved from the Protein Data Bank (PDB ID: 2Z64). Molecular docking of the synthesized hybrid peptides with MD2 was performed using the HDOCK web server (http://hdock.phys.hust.edu.cn/, (accessed on 7 January 2025)). The structural validity of the docking complexes was assessed using the Ramachandran plot tool available at https://saves.mbi.ucla.edu/, (accessed on 7 January 2025). Based on the docking scores, the top ten hybrid peptide candidates were selected, synthesized, and further evaluated in vitro.

### 4.3. Cell Culture

RAW 264.7 mouse macrophages were purchased from the Shanghai Cell Bank, Institute of Cell Biology, Chinese Academy of Sciences, and cultured in Dulbecco’s Modified Eagle’s Medium (DMEM; HyClone, UT, USA), supplemented with 10% fetal bovine serum (Procell, Wuhan, China) and 1% penicillin–streptomycin (Solarbio, Beijing, China). Cells were maintained at 37 °C in a humidified environment with 5% CO_2_ and 95% air and passaged daily.

IPEC-J2 cells were obtained from Procell, cultured under the same conditions as RAW 264.7 cells, with medium changes every other day, and passaged every 3–4 days.

The RAW-Lucia NF-κB and IPEC-J2-Lucia ARE cell lines, stably transfected in-house, were maintained under identical conditions to their wild-type counterparts.

### 4.4. Construction of Luciferase Reporter Gene Stable Cell Lines and Luciferase Reporter Assay

The ARE sequences and NF-κB element sequences are referenced in [52,54]. Based on the sequence information, plasmids pCD-ARE-puro and pCDH-NF-κB-puro were constructed. These plasmids, along with pMD2.G and psPAX2, were transfected into HEK293T cells using a three-plasmid virus packaging system to produce viruses. The virus supernatants were then used to infect IPEC-J2 or RAW 264.7 cells, followed by positive clone screening with Puromycin. For the testing of stable cell lines, tBHQ was used for IPEC-J2-Lucia ARE cells and LPS was used for RAW-Lucia NF-κB cells.

Candidate peptides (100 μg/mL) were incubated with RAW-Lucia NF-κB or IPEC-J2-Lucia ARE cells (3 × 10⁵ cells/well) in 96-well plates for 12 h. LPS (100 ng/mL) was used as the positive control for the RAW-Lucia NF-κB cells and tBHQ (10 μM) was used as the positive control for the IPEC-J2-Lucia ARE cells. Subsequently, 15 μL of the culture supernatant was mixed with 50 μL of detection buffer (50 mM HEPES, pH 7.0; 50 mM NaCl; 0.05% CHAPS; 10 mM EDTA; and 1 μM coelenterazine). Luminescence was measured using a multimode plate reader, and relative luminescence units (RLUs) were calculated.

### 4.5. Cytotoxicity Assay

The cytotoxicity of the hybrid and parent peptides on RAW 264.7 cells was evaluated using a CCK-8 assay (Solarbio, Beijing, China). RAW 264.7 cells (3 × 10⁵ cells/well) were seeded into 96-well plates and incubated for 12 h, followed by treatment with various concentrations of hybrid peptides (0, 10, 20, 30, 40, 50, 60, 70, 80, 90, and 100 μg/mL) for 24 h. Afterward, 10 μL of CCK-8 reagent was added to each well and incubated at 37 °C for 0.5–2 h. Absorbance was measured at 450 nm, and cell viability was calculated using the following formula:

$$Cell\;viability\left(\%\right)=\frac{A_{S}-A_{B}}{A_{C}-A_{B}}\times 100\%$$

where *A_S_* is the absorbance of wells containing cells, CCK-8, and peptides; *A_B_* is the absorbance of wells containing medium and CCK-8 without cells; and *A_C_* is the absorbance of wells containing cells and CCK-8 without peptides.

### 4.6. Hemolysis Assay

Fresh sheep red blood cells (RBCs) (Solarbio, Beijing, China) were mixed with an equal volume of PBS and centrifuged at 800× *g* for 5 min. The supernatant was discarded and the RBCs were resuspended in PBS. This washing process was repeated until the supernatant became clear. An 8% RBC suspension was prepared by mixing the cells with PBS. Subsequently, 10 μL of various peptide concentrations (0, 10, 20, 30, 40, 50, 60, 70, 80, 90, and 100 μg/mL) were added to 96-well plates, followed by 90 μL of the RBC suspension. The plates were incubated at 37 °C for 1 h and centrifuged at 800× *g* for 5 min. The supernatant was collected for detection. Absorbance of the supernatant was measured at 414 nm. Triton X-100 (0.1%) served as the positive control and PBS was used as the negative control. Hemolysis was calculated using the following formula:

$$Hemolysis\;rate\left(\%\right)=\frac{A-A_{0}}{A_{1}}\times 100\%$$

where *A* is the absorbance of the sample, *A*_0_ is the absorbance of the negative control, and *A*_1_ is the absorbance of the positive control.

### 4.7. Enzyme-Linked Immunosorbent Assay (ELISA)

RAW 264.7 macrophages (2 × 10^6^ cells/well) were seeded in 6-well plates and incubated for 12 h. Hybrid peptides and parent peptides were added to achieve a final concentration of 100 μg/mL, LPS (100 ng/mL) as a positive control. After 24 h of incubation, the culture supernatants were collected, and the levels of TNF-α, IL-6, and IL-1β were measured using ELISA kits according to the manufacturer’s instructions (Solarbio, Beijing, China).

### 4.8. H_2_O_2_-Induced Oxidative Damage Model in HepG2 Cells

HepG2 cells (2 × 10^5^ cells/well) were seeded into 96-well plates and incubated overnight at 37 °C with 5% CO_2_. The supernatant was removed, and hybrid or parent peptides were added at a final concentration of 100 ng/mL for 2 h. H_2_O_2_ (Sigma-Aldrich, St. louis, MO, USA) was then added to a final concentration of 650 μM and the cells were further incubated for 6 h. Cell viability was assessed using the CCK-8 assay.

### 4.9. Statistical Analysis

Statistical analysis was performed using GraphPad Prism v9.0. Data were analyzed using Student’s *t*-test for comparisons between two groups. All data are expressed as the mean ± SD of at least three independent experiments. Statistical significance was defined as *p* ≤ 0.05. * *p* ≤ 0.05, ** *p* ≤ 0.01, *** *p* ≤ 0.001, and **** *p* ≤ 0.0001.

## 5. Conclusions

This study demonstrates the successful design and evaluation of hybrid peptides that can modulate both immune responses and oxidative stress. By utilizing peptide hybridization, we generated multifunctional peptides with combined immunomodulatory and antioxidant properties. The top-performing peptide, VA, exhibited significant activity in enhancing immune responses and protecting against oxidative stress, making it a promising candidate for further therapeutic development. Our approach not only expands the potential of peptide-based therapies but also provides a platform for the creation of multifunctional peptides with therapeutic applications in cancer, autoimmune disorders, and oxidative stress-related conditions.

## Figures and Tables

**Table 1:** Sequence information of parent peptides.

Peptide	Sequence	Abbreviation
TP5	RKDVY	P
Tα1-a	KEKKEVVE	Ta
Tα1-b	EVVEEA	Tb
Aβ	DAEFRHDSGYEVHHQKLVFFAEDVGSNKGAIIGLMVG	A
RP5	RGPPP	R
PH	TQIDKVVHFDKLPGF	H
YG9	YGPSSYGYG	Y
MMO	QLNWD	M
VLP	VLPVPQK	V

**Table 2:** Sequence information of hybrid peptides.

	Peptide Name	Sequence	Residue Number	Molecular Weight (Da)	Water Solubility	Toxicity Prediction
1	PR	RKDVYRGPPP	10	1184.35	Good	non
2	PV	RKDVYVLPVPQK	12	1441.71	Good	non
3	PH	RKDVYTQIDKVVHFDKLPGF	20	2405.75	Good	non
4	PY	RKDVYYGPSSYGYG	14	1611.71	Good	non
5	PM	RKDVYQLNWD	10	1336.45	Good	non
6	RP	RGPPPRKDVY	10	1184.35	Good	non
7	VP	VLPVPQKRKDVY	12	1441.71	Good	non
8	HP	TQIDKVVHFDKLPGFRKDVY	20	2405.75	Good	non
9	YP	YGPSSYGYGRKDVY	14	1611.71	Good	non
10	MP	QLNWDRKDVY	10	1336.45	Good	non
11	TaR	KEKKEVVERGPPP	13	1492.72	Good	non
12	TaV	KEKKEVVEVLPVPQK	15	1750.08	Good	non
13	TaH	KEKKEVVETQIDKVVHFDKLPGF	23	2714.12	Good	non
14	TaY	KEKKEVVEYGPSSYGYG	17	1920.08	Good	non
15	TaM	KEKKEVVEQLNWD	13	1644.82	Good	non
16	RTa	RGPPPKEKKEVVE	13	1492.72	Good	non
17	VTa	VLPVPQKKEKKEVVE	15	1750.08	Good	non
18	HTa	TQIDKVVHFDKLPGFKEKKEVVE	23	2714.12	Good	non
19	YTa	YGPSSYGYGKEKKEVVE	17	1920.08	Good	non
20	MTa	QLNWDKEKKEVVE	13	1644.82	Good	non
21	TbR	EVVEEARGPPP	11	1179.28	Good	non
22	TbV	EVVEEAVLPVPQK	13	1436.64	Good	non
23	TbH	EVVEEATQIDKVVHFDKLPGF	21	2400.68	Good	non
24	TbY	EVVEEAYGPSSYGYG	15	1606.64	Good	non
25	TbM	EVVEEAQLNWD	11	1331.38	Good	non
26	RTb	RGPPPEVVEEA	11	1179.28	Good	non
27	VTb	VLPVPQKEVVEEA	13	1436.64	Good	non
28	HTb	TQIDKVVHFDKLPGFEVVEEA	21	2400.68	Good	non
29	YTb	YGPSSYGYGEVVEEA	15	1606.64	Good	non
30	MTb	QLNWDEVVEEA	11	1331.38	Good	non
31	AR	DAEFRHDSGYEVHHQKLVFFAEDVGSNKGAIIGLMVGRGPPP	42	4579.05	Good	non
32	AV	DAEFRHDSGYEVHHQKLVFFAEDVGSNKGAIIGLMVGVLPVPQK	44	4836.42	Good	non
33	AH	DAEFRHDSGYEVHHQKLVFFAEDVGSNKGAIIGLMVGTQIDKVVHFDKLPGF	52	5800.44	Good	non
34	AY	DAEFRHDSGYEVHHQKLVFFAEDVGSNKGAIIGLMVGYGPSSYGYG	46	5006.41	Good	non
35	AM	DAEFRHDSGYEVHHQKLVFFAEDVGSNKGAIIGLMVGQLNWD	42	4731.15	Good	non
36	RA	RGPPPDAEFRHDSGYEVHHQKLVFFAEDVGSNKGAIIGLMVG	42	4579.05	Good	non
37	VA	VLPVPQKDAEFRHDSGYEVHHQKLVFFAEDVGSNKGAIIGLMVG	44	4836.42	Good	non
38	HA	TQIDKVVHFDKLPGFDAEFRHDSGYEVHHQKLVFFAEDVGSNKGAIIGLMVG	52	5800.44	Good	non
39	YA	YGPSSYGYGDAEFRHDSGYEVHHQKLVFFAEDVGSNKGAIIGLMVG	46	5006.41	Good	non
40	MA	QLNWDDAEFRHDSGYEVHHQKLVFFAEDVGSNKGAIIGLMVG	42	4731.15	Good	non

**Table 3:** HDOCK docking scores of hybrid peptides.

	Peptide	Sequence	HDOCK Score
1	YP	YGPSSYGYGRKDVY	−309.2
2	PY	RKDVYYGPSSYGYG	−229.8
3	YTa	YGPSSYGYGKEKKEVVE	−207.6
4	VA	VLPVPQKDAEFRHDSGYEVHHQKLVFFAEDVGSNKGAIIGLMVG	−203.7
5	PR	RKDVYRGPPP	−198.1
6	MP	QLNWDRKDVY	−194.6
7	RP	RGPPPRKDVY	−193.1
8	YTb	YGPSSYGYGEVVEEA	−191.2
9	TbY	EVVEEAYGPSSYGYG	−191
10	TaY	KEKKEVVEYGPSSYGYG	−189.6
11	TaV	KEKKEVVEVLPVPQK	−184
12	PV	RKDVYVLPVPQK	−182.3
13	PH	RKDVYTQIDKVVHFDKLPGF	−179.4
14	VTa	VLPVPQKKEKKEVVE	−177.2
15	TaH	KEKKEVVETQIDKVVHFDKLPGF	−176.6
16	PM	RKDVYQLNWD	−176.3
17	HTa	TQIDKVVHFDKLPGFKEKKEVVE	−175.3
18	MTb	QLNWDEVVEEA	−175.1
19	TbH	EVVEEATQIDKVVHFDKLPGF	−174.7
20	AH	DAEFRHDSGYEVHHQKLVFFAEDVGSNKGAIIGLMVGTQIDKVVHFDKLPGF	−172.8
21	HTb	TQIDKVVHFDKLPGFEVVEEA	−172.6
22	TbV	EVVEEAVLPVPQK	−169.9
23	TbM	EVVEEAQLNWD	−169.2
24	VP	VLPVPQKRKDVY	−167.6
25	VTb	VLPVPQKEVVEEA	−167.5
26	HP	TQIDKVVHFDKLPGFRKDVY	−167.3
27	AY	DAEFRHDSGYEVHHQKLVFFAEDVGSNKGAIIGLMVGYGPSSYGYG	−163.9
28	YA	YGPSSYGYGDAEFRHDSGYEVHHQKLVFFAEDVGSNKGAIIGLMVG	−163.8
29	HA	TQIDKVVHFDKLPGFDAEFRHDSGYEVHHQKLVFFAEDVGSNKGAIIGLMVG	−162.2
30	AV	DAEFRHDSGYEVHHQKLVFFAEDVGSNKGAIIGLMVGVLPVPQK	−161.9
31	AM	DAEFRHDSGYEVHHQKLVFFAEDVGSNKGAIIGLMVGQLNWD	−158.1
32	TaM	KEKKEVVEQLNWD	−157.8
33	TbR	EVVEEARGPPP	−156.2
34	MA	QLNWDDAEFRHDSGYEVHHQKLVFFAEDVGSNKGAIIGLMVG	−154.4
35	TaR	KEKKEVVERGPPP	−153.8
36	MTa	QLNWDKEKKEVVE	−151.2
37	RTa	RGPPPKEKKEVVE	−144.8
38	AR	DAEFRHDSGYEVHHQKLVFFAEDVGSNKGAIIGLMVGRGPPP	−140.4
39	RTb	RGPPPEVVEEA	−136.8
40	RA	RGPPPDAEFRHDSGYEVHHQKLVFFAEDVGSNKGAIIGLMVG	−131.3

**Table 4:** Ramachandran plot analysis of the conformational plausibility of docking complexes.

	Peptide Name	Sequence	Core	Allow	Core+ Allow	Gener	Disall
1	PR	RKDVYRGPPP	83.1	16.1	99.2	0.0	0.8
2	PV	RKDVYVLPVPQK	84.3	14.2	98.5	0.0	1.5
3	PH	RKDVYTQIDKVVHFDKLPGF	83.7	15.6	99.3	0.7	0.0
4	PY	RKDVYYGPSSYGYG	84.4	15.6	100.0	0.0	0.0
5	PM	RKDVYQLNWD	83.5	15.7	99.2	0.8	0.0
6	RP	RGPPPRKDVY	85.4	14.6	100.0	0.0	0.0
7	VP	VLPVPQKRKDVY	83.6	16.4	100.0	0.0	0.0
8	HP	TQIDKVVHFDKLPGFRKDVY	82.2	17.8	100.0	0.0	0.0
9	YP	YGPSSYGYGRKDVY	82.7	17.3	100.0	0.0	0.0
10	MP	QLNWDRKDVY	85.0	15.0	100.0	0.0	0.0
11	TaR	KEKKEVVERGPPP	85.8	14.2	100.0	0.0	0.0
12	TaV	KEKKEVVEVLPVPQK	82.5	16.8	99.3	0.7	0.0
13	TaH	KEKKEVVETQIDKVVHFDKLPGF	84.1	15.2	99.3	0.7	0.0
14	TaY	KEKKEVVEYGPSSYGYG	84.7	15.3	100.0	0.0	0.0
15	TaM	KEKKEVVEQLNWD	85.4	14.6	100.0	0.0	0.0
16	RTa	RGPPPKEKKEVVE	84.9	15.1	100.0	0.0	0.0
17	VTa	VLPVPQKKEKKEVVE	84.7	13.9	98.6	1.5	0.0
18	HTa	TQIDKVVHFDKLPGFKEKKEVVE	85.5	14.5	100.0	0.0	0.0
19	YTa	YGPSSYGYGKEKKEVVE	84.6	15.4	100.0	0.0	0.0
20	MTa	QLNWDKEKKEVVE	86.2	13.8	100.0	0.0	0.0
21	TbR	EVVEEARGPPP	84.0	16.0	100.0	0.0	0.0
22	TbV	EVVEEAVLPVPQK	85.2	14.1	99.3	0.7	0.0
23	TbH	EVVEEATQIDKVVHFDKLPGF	86.0	14.0	100.0	0.0	0.0
24	TbY	EVVEEAYGPSSYGYG	82.9	15.5	98.4	1.6	0.0
25	TbM	EVVEEAQLNWD	85.2	14.1	99.3	0.8	0.0
26	RTb	RGPPPEVVEEA	85.5	14.5	100.0	0.0	0.0
27	VTb	VLPVPQKEVVEEA	85.2	14.8	100.0	0.0	0.0
28	HTb	TQIDKVVHFDKLPGFEVVEEA	86.8	13.2	100.0	0.0	0.0
29	YTb	YGPSSYGYGEVVEEA	83.6	15.6	99.2	0.8	0.0
30	MTb	QLNWDEVVEEA	85.9	14.1	100.0	0.0	0.0
31	AR	DAEFRHDSGYEVHHQKLVFFAEDVGSNKGAIIGLMVGRGPPP	85.8	14.2	100.0	0.0	0.0
32	AV	DAEFRHDSGYEVHHQKLVFFAEDVGSNKGAIIGLMVGVLPVPQK	86.1	13.9	100.0	0.0	0.0
33	AH	DAEFRHDSGYEVHHQKLVFFAEDVGSNKGAIIGLMVGTQIDKVVHFDKLPGF	86.2	13.8	100.0	0.0	0.0
34	AY	DAEFRHDSGYEVHHQKLVFFAEDVGSNKGAIIGLMVGYGPSSYGYG	85.5	14.5	100.0	0.0	0.0
35	AM	DAEFRHDSGYEVHHQKLVFFAEDVGSNKGAIIGLMVGQLNWD	85.4	14.6	100.0	0.0	0.0
36	RA	RGPPPDAEFRHDSGYEVHHQKLVFFAEDVGSNKGAIIGLMVG	85.7	14.3	100.0	0.0	0.0
37	VA	VLPVPQKDAEFRHDSGYEVHHQKLVFFAEDVGSNKGAIIGLMVG	86.1	13.9	100.0	0.0	0.0
38	HA	TQIDKVVHFDKLPGFDAEFRHDSGYEVHHQKLVFFAEDVGSNKGAIIGLMVG	85.5	14.5	100.0	0.0	0.0
39	YA	YGPSSYGYGDAEFRHDSGYEVHHQKLVFFAEDVGSNKGAIIGLMVG	84.6	15.4	100.0	0.0	0.0
40	MA	QLNWDDAEFRHDSGYEVHHQKLVFFAEDVGSNKGAIIGLMVG	86.2	13.8	100.0	0.0	0.0

Note: Amino acid residues that fall within the core and allowed regions (maximum allowable areas) account for over 90% of the residues in the protein, indicating that the model adheres to the rules of stereochemistry.

## Data Availability

Data are contained within the article.

## Supplementary Materials

The following supporting information can be downloaded at: https://www.mdpi.com/article/10.3390/ijms26020505/s1.

## Abbreviations

The following abbreviations are used in this manuscript:

TLR4	Toll-like receptor 4
MD2	Myeloid differentiation protein-2
LPS	Lipopolysaccharides
NF-κB	Nuclear factor kappa-B
ARE	Antioxidant response elements
Keap1	Kelch-like ECH-associated protein 1
Nrf2	Nuclear factor erythroid 2-related factor 2
TNF-α	Tumor necrosis factor-α
IL-6	Interleukin-6
IL-1β	Interleukin-1β
