# Supplementary material for: Hybridization Design and High-Throughput Screening of Peptides with Immunomodulatory and Antioxidant Activities
Source: Int J Mol Sci. 2025 Jan 9;26(2):505. doi: 10.3390/ijms26020505 (PMC11764585; doi:10.3390/ijms26020505)
Supplement: Supplementary file 1 [file ijms-26-00505-s001.zip › Figure S1.pptx]

## Slide 1
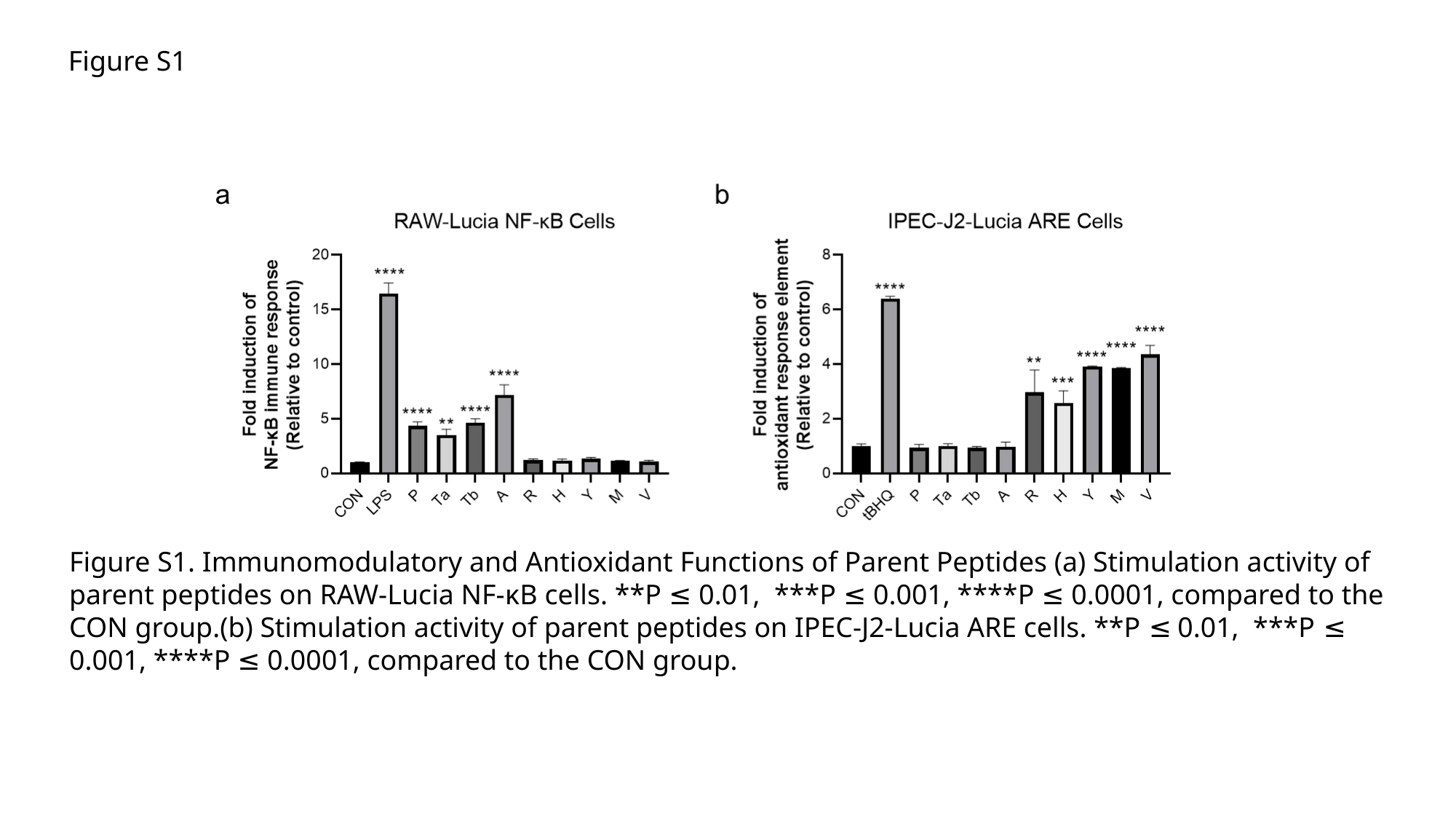

Figure S1
Figure S1. Immunomodulatory and Antioxidant Functions of Parent Peptides (a) Stimulation activity of parent peptides on RAW-Lucia NF-κB cells. **P ≤ 0.01, ***P ≤ 0.001, ****P ≤ 0.0001, compared to the CON group.(b) Stimulation activity of parent peptides on IPEC-J2-Lucia ARE cells. **P ≤ 0.01, ***P ≤ 0.001, ****P ≤ 0.0001, compared to the CON group.
