# Supplementary material for: Hybridization Design and High-Throughput Screening of Peptides with Immunomodulatory and Antioxidant Activities
Source: Int J Mol Sci. 2025 Jan 9;26(2):505. doi: 10.3390/ijms26020505 (PMC11764585; doi:10.3390/ijms26020505)
Supplement: Supplementary file 1 [file ijms-26-00505-s001.zip › Synthesis peptides MS results.zip!/MP-MS.pdf]

MASS SPECTROMETRY REPORT

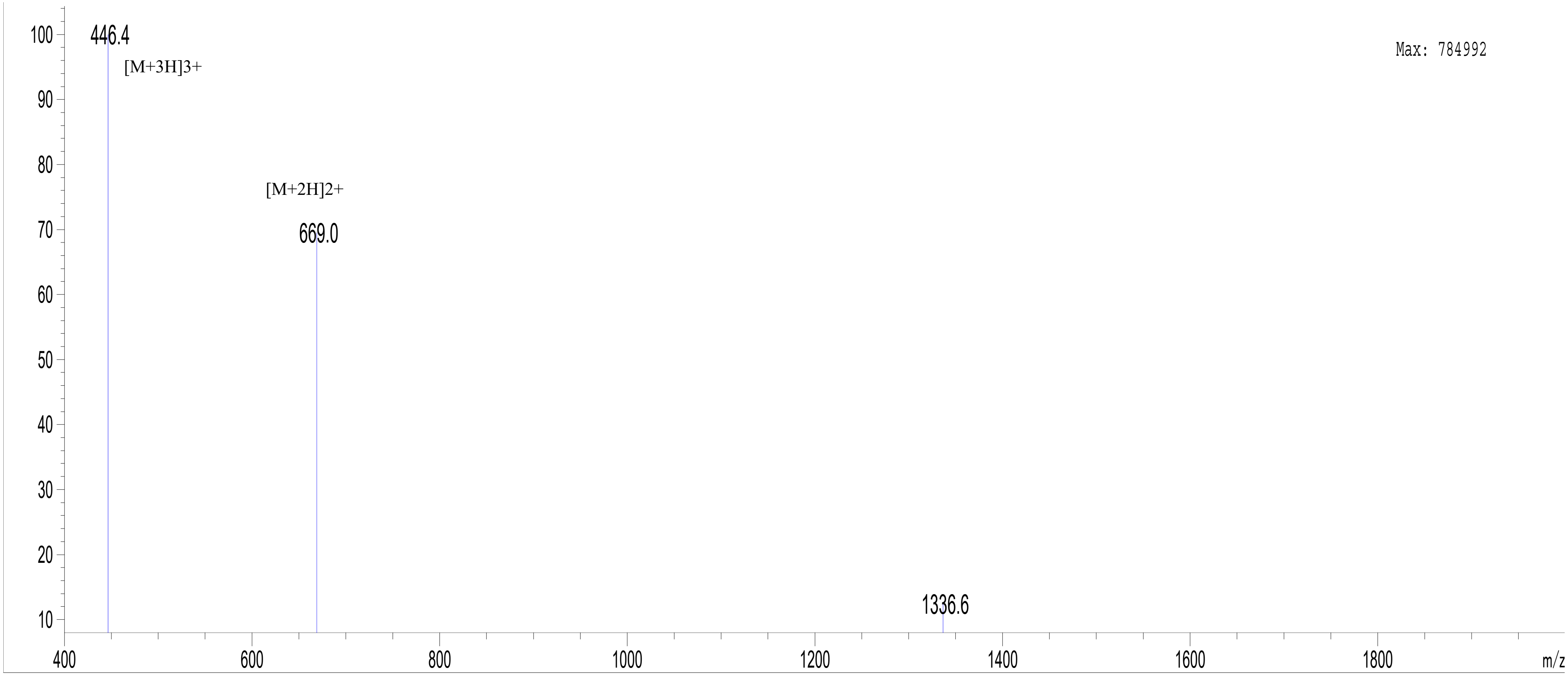

Sample Description		Instrument	Agilent-6125B		
Analyzed date:	2023-04-25	Probe:	ESI	Probe Bias:	＋4.5kv
Analyst:	YU	Nebulizer Gas Flow:	1.5L/min	Detector:	1.5kv
Sample:	MP	CDL:	-20.0v	T. Flow:	0.2ml/min
M.W.:	1336.45	CDL Temp.:	250 ^{o}C	B. Conc.:	50%H2O/50%ACN
Lot. No.:	P230328-SJ1067168	Block Temp.:	200 ^{o}C		
